# Uterine prolapse and associated factors among reproductive-age women in south-west Ethiopia: A community-based cross-sectional study

**DOI:** 10.1371/journal.pone.0262077

**Published:** 2022-01-21

**Authors:** Abebe Sorsa Badacho, Mengistu Auro Lelu, Zegeye Gelan, Deginesh Dawit Woltamo

**Affiliations:** School of Public Health, College of Health Sciences and Medicine, Wolaita Sodo University, Wolaita Sodo, Ethiopia; Weill Cornell Medical College, UNITED STATES

## Abstract

**Background:**

Uterine prolapse is an important but neglected public health problem that causes maternal morbidity and mortality in women of reproductive age in low- and middle-income countries, including Ethiopia. However, little data are available concerning uterine prolapse in Ethiopia. The objective of this study was to assess the prevalence of and factors associated with uterine prolapse in women of reproductive age in Ethiopia.

**Methods:**

A community-based cross-sectional study was conducted in Loma Woreda, Dawro, south-west Ethiopia, in November and December 2019. Four hundred and twenty-two randomly selected women of reproductive age participated in the study. Face-to-face interviews with a pre-structured questionnaire collected data, and diagnoses were made clinically. Epi Data v3.2.1 and SPSS v24 were used for data entry, processing, and analysis. Binary logistic regression was used to determine associations between dependent and independent variables. Variables with P-values less than 0.25 in bivariate logistic regression were further examined using multivariate logistic regression to investigate associations between the dependent variable and independent variables.

**Results:**

The mean age of respondents was 35.4 ±7.994 years. The prevalence of symptomatic and anatomical uterine prolapse was 6.6% (28) and 5.9% (25), respectively. The prevalence of anatomical prolapse was used as a reference when determining associated factors. Age at first marriage (Adjusted Odd Ratio (AOR): 0.25, 95%CI 0.07, 0.89), place of delivery (AOR: 3.33, 95%CI 1.21, 9.13), birth attendant-assisted delivery (AOR 0.21; 95%CI 0.06, 0.71), and history of abortion (AOR: 2.94, 95%CI 1.08, 7.97) were found significantly and independently associated with the prevalence of uterine prolapse.

**Conclusion:**

Uterine prolapse is common in women of reproductive age. Age at first marriage, place of delivery, birth attendant-assisted delivery, and history of abortion were independent predictors of the prevalence of uterine prolapse. We recommend that the health system link primary health care to hospital-set for uterine prolapse treatment programs. Health institution delivery should be encouraged by the local government. Early marriage and unwanted pregnancy need to be prevented through appropriate strategies.

## Introduction

Uterine prolapse (UP), also known as pelvic organ prolapse (POP) and genital prolapse, describes the descent of the uterus from its normal anatomical confines to positions within or outside the vaginal introitus. UP occurs secondary to weakened pelvic muscles that can no longer support the appropriate positioning of the pelvic organs and can be accompanied by different prolapse symptoms like a feeling of heaviness and sexual, urinary, and bowel dysfunction [1].

UP is the most common gynecological health problem contributing to maternal morbidity and mortality in women of reproductive age in developing countries. It leads to varying degrees of physical disability, including an inability to work, difficulties in walking or standing up, difficulties in urinating or defecating, painful intercourse, increased social stigma, and economic deprivation. UP can also affect women’s mental health and can be fatal if left untreated [1–3]. Due to stigma in low-income countries, women affected by UP often hide their condition, do not seek help, and live with the disease and its complications for long periods [4].

The worldwide prevalence of UP has been reported to be around 9%. However, in low and middle-income countries (LMICs), it is estimated to be nearly 20%, and estimates vary widely (3.4–56.4%) [5]. The prevalence based on symptoms is 3–6% and up to 50% when defined by vaginal checkups [6].

The burden of UP in low-income countries is expected to be worse than that of developed countries, given the low level of awareness of women in developing countries [4, 7, 8].

Major risk factors associated with UP are adolescent pregnancy, lack of rest during and immediately after pregnancy, carrying heavy loads, delivery by unskilled birth attendants, poor nutrition, frequent pregnancies and pregnancies close together, prolonged and obstructed labor, and weakening of pelvic muscles as a result of aging or other medical problems [3, 9].

UP is, therefore, an important but one of the most neglected public health problems in LMICs, including Ethiopia, where there is little literature regarding its prevalence [10, 11]. Indeed, there have been no published population-based studies on UP in Ethiopia, although reports from individual hospitals suggest a high burden of UP among women at gynecological outpatient clinics and wards [5, 9].

**Fig 1:**
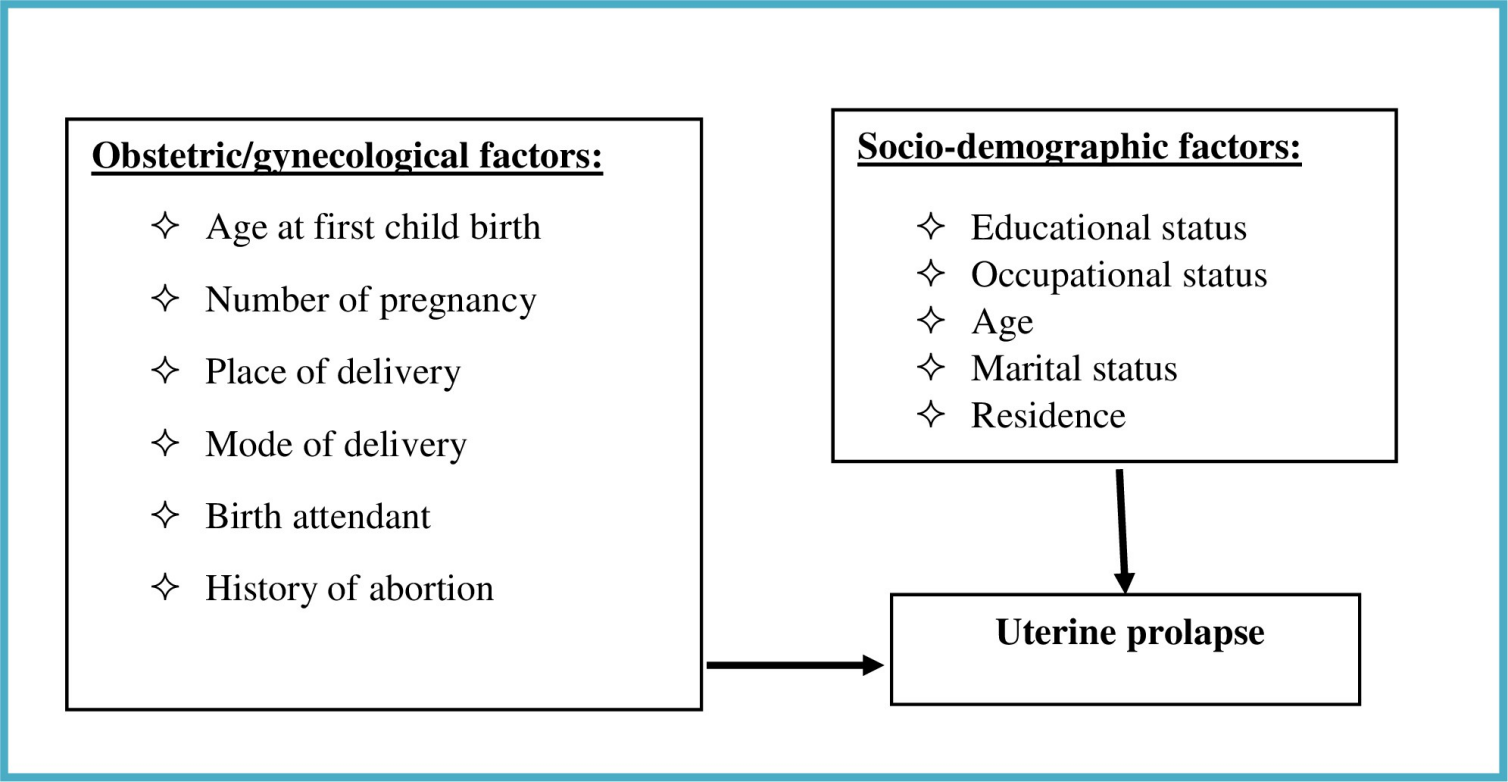
Conceptual framework of the study developed from reviewing the literature.

The high numbers of women potentially affected by UP and the paucity of locally generated evidence on the magnitude and associated factors of UP to design appropriate prevention strategies [10, 12], here we assessed the prevalence of and factors associated with uterine prolapse in women of reproductive age in Ethiopia (Fig 1).

## Materials and methods

### Study design and period

A community based cross-sectional study was conducted in November and December 2019.

### Source and study population

The source or target population of the study was all women of the reproductive age group who had a history of at least one delivery, while the study population was all women randomly selected.

### Inclusion criteria

All ever-married and single women of reproductive age group greater and equal 18 years who had a history of at least one delivery were included. Pregnant women were excluded.

### Sample size determination and sampling procedure

The sample size was calculated to ensure that the two-sided 95% confidence interval (CI) for the estimated prevalence will be within +/- 0.05 by using a single population formula with a proportion of 0.5.

Four hundred twenty-two randomly selected women were involved in the study using a household as a sampling frame. From the total 28 kebeles in the Loma district, 30% of kebeles, i.e., eight kebeles, which is the lowest administrative unit in Ethiopia, were selected by the lottery method. We raffled eight areas of the Loma district and then selected the sample by a simple random sampling in these areas. The assumption was to divide the total estimated sample size to the households of each kebele according to the proportion they contribute to the total study subjects. We allocated sample proportion for the selected eight kebeles based on sample size. Out of an estimated 422 participants, the sample size was adjusted proportionally for the selected eight kebeles. Then, married women of reproductive age were selected by random sampling method, using a married reproductive age group list from a registration book of health post as provided by Health Extension Workers (HEWs) working at Health Post in each respective kebele.” Table 1.

**Table 1:** Proportional allocation of study participants from each kebele in the Loma woreda, Dawuro Zone, Ethiopia 2020.

Kebeles	Kebele 1	Kebele 2	Kebele 3	Kebele 4	Kebele 5	Kebele 6	Kebele7	Kebele 8
Total number of married Reproductive age group in households	673	735	812	741	620	65	705	720
Number of women participated in the study	50	55	61	55	46	48	53	54

### Data collection

Eight trained midwives collected data through face-to-face interviews at participants’ homes supervised by two BSc-qualified Nurse Supervisors.

### Quality assurance

Data collectors and supervisors were trained for three days about the study, communication, respecting the cultural norms of women, detailed review procedures, the informed consent process, and administration of the study questionnaire.

Before collecting the actual data, 5% of the total sample size was pretested, and necessary corrections to the questionnaires were made accordingly. The questionnaire was translated and back-translated from English to Amharic and back to English to check the consistency. At the end of each data collection day, data were checked for completeness and consistency and discussed with the research assistants.

### Data collection instrument and measurement

A structured questionnaire was developed from similar uterine prolapse prevalence studies. The interview question was composed of three main sections. The first two were phase 1, and the third was considered as phase two:

1. socio-demographic variables and obstetric and gynecologic history (14 questions);
2. questions regarding symptoms of uterine prolapsed (6 questions);
3. confirming by vaginal examination whether the women who reported symptomatic prolapse had anatomical prolapse or not and prolapse staging of the present.

Symptomatic POP was assessed by two questions previously used in other studies: do you have a (1) feeling of bulging/pressure or something that seems to be coming down through the vagina or (2) visible mass protruding via the vagina? A woman who had experienced one or both of these problems in the past year was considered as having symptoms of UP, and further questions assessed the duration and associated symptoms. An indication of prolapse based on the questionnaire was referred to as symptomatic prolapse. Women who reported the symptoms of uterine prolapse were referred to Tercha Zonal hospital for pelvic examination to further identify the anatomical prolapse and its stage. A gynaecologist performed pelvic examination using the Pelvic Organ Prolapse Quantification (POP-Q) system at the hospital along with care and treatment.

### Data analysis

Data entry and analysis were conducted using Epi Data v3.2.1 and SPSS v25. Data were cleaned before analysis. The means, frequencies, and percentages were calculated, and bivariate and multivariable logistic regression was carried out to examine the relationships between the independent and dependent variables. Variables with P-values less than 0.25 in bivariate logistic regression were further examined using multivariate logistic regression to investigate associations between the dependent variable and independent variables. Adjusted odds ratio (AOR) was used and a P-value <0.05 was considered statistically significant.

## Results

### Socio-demographic characteristics of study participants

Four hundred and twenty-two women participated in the study, a response rate of 100%. The mean age (+SD) of respondents was 35.4 (±7.99) years, and the mean ages (+SD) at first marriage and first childbirth were 18.14 (±2.151) and 19.94 (±2.921), respectively. The mean numbers of pregnancies and childbirths were 3.94 and 3.80, respectively. Three hundred and sixty-six women (86.5%) were married; over three-quarters (78%) of participants were rural residents. Over half of the participants were unable to read and write or had only primary education (235; 55.7%). Around three-quarters of respondents were housewives (313, 74.2%). More than three fourth of respondents (322, 76.3%) were protestant religion followers (**Table 2**).

**Table 2:** Socio-demographic characteristics of study participants in the Loma Woreda, Dawuro Zone, Ethiopia 2020 (n = 422).

Variable	Category	Frequency	Percentage
Marital status	Married	366	86.72
	Divorced	30	7.1
	Widowed	26	6.16
Residence	Urban	93	22.0
	Rural	329	78.0
Educational status	Unable to read and write	91	21.6
	Primary school	144	34.1
	Secondary school	128	30.3
	Higher education	59	14.0
Occupational status	House wife	313	74.2
	Government worker	65	15.4
	Merchant	36	8.5
	Others	8	1.9
Religion	Orthodox	93	22.0
	Protestant	322	76.3
	Others*	7	1.7

*Others, musilms, catholics.

### Obstetric and gynecological variables

Half of the participants (217, 51.4%) had a history of home delivery only, while 122 (28.9%) had a history of delivery in a healthcare institution. The largest proportion of study subjects, 89.3% (377), had a history of normal vaginal delivery, while 3.6% and 2.8% had had caesarian sections and operative deliveries, respectively. Only a third of study participants (141) had a history of delivery assisted by health personnel. One hundred and fifty-three (36.3%) and one in ten (10%) women’s deliveries were assisted by family/relatives and traditional birth attendants. One in ten of (10.2%) participants had a history of abortion (**Table 3**).

**Table 3:** Obstetric and gynecological characteristics of study participants in Loma Woreda, Dawuro Zone, Ethiopia 2020 (n = 422).

Variable	Category	Frequency	Percentage
Age at first marriage	Less than 18 years	252	59.7
	18 and above years	170	40.3
Age at first childbirth	Less than 18 years	113	26.8
	18 and above years	309	73.2
Number of pregnancies	Grand multipara	158	37.4
	Primipara	59	14.0
	Multipara	205	48.6
Place of delivery	Home delivery	217	51.4
	Health institution	122	28.9
	Both home delivery and health institution	83	19.7
Mode of delivery	Normal vaginal delivery	377	89.3
	Operative delivery	12	2.8
	Caesarian section	15	3.6
	Two or more of the above	18	4.3
Birth attendant	Health personnel	141	33.4
	Traditional birth attendant	42	10.0
	Family or relatives	153	36.3
	Two or more of the above	86	20.4
History of abortion	No	379	89.8
	Yes	43	10.2

### Prevalence of uterine prolapse

Initial screening with midwives, 28 (6.6%) subjects suspected UP and were referred to doctor examination, and only 25(5.9%) participants confirmed that they had UP. All of these 28 women reported a feeling of bulging, pressure, or something coming down from the vagina and nine had a visible mass protruding from the vagina (2.1% of the total study subjects). Of the 28 women who reported symptomatic uterine prolapse, about two-third of (67.86%) had either a feeling of bulging/something coming down in the vagina or a visible mass protruding from the vagina, whereas the other nine (32.14%) reported both bulging and a visible mass protruding from the vagina.

**Fig 2:**
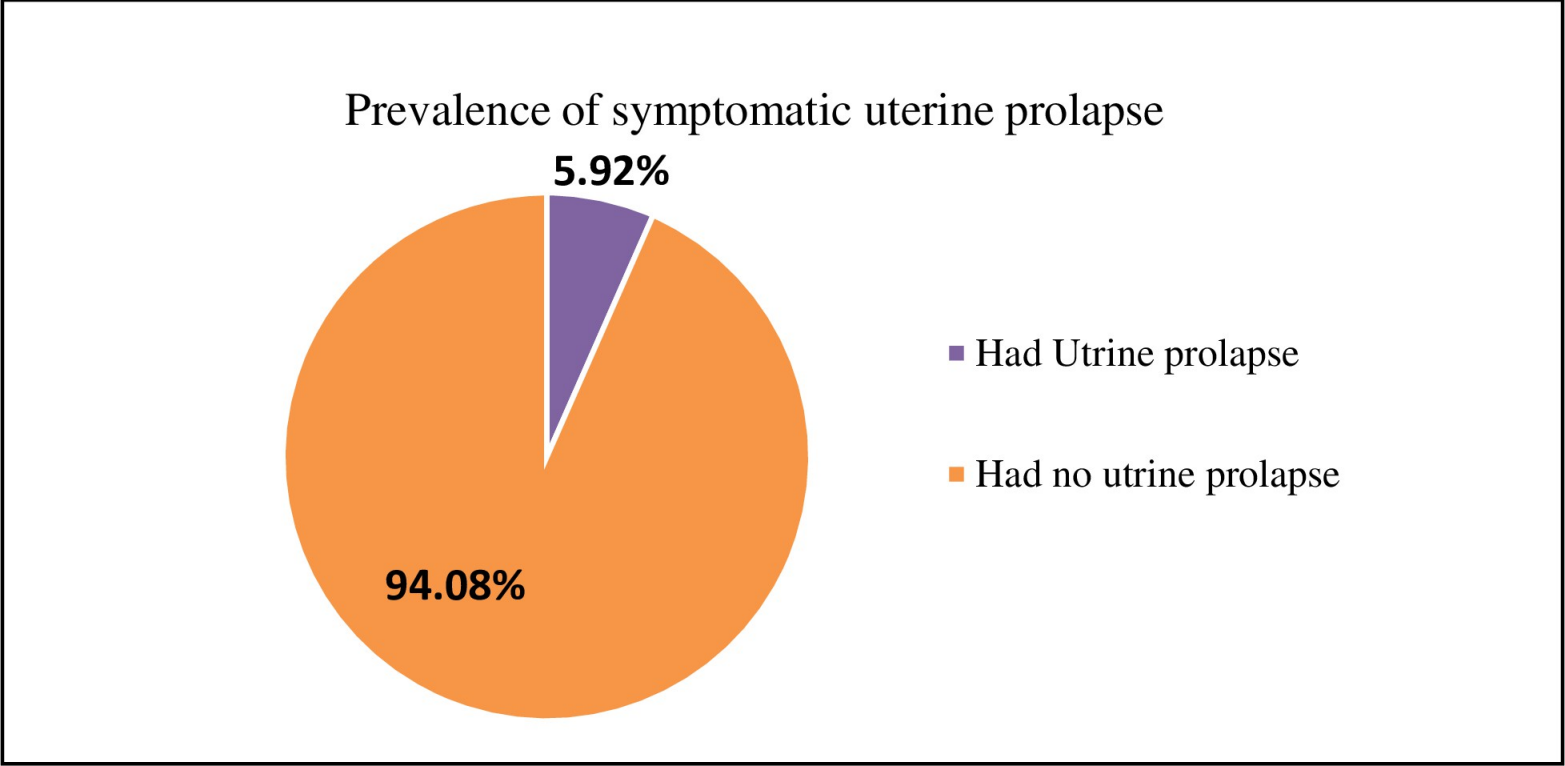
Prevalence of symptomatic uterine prolapse in Loma Woreda, Dawuro Zone, Ethiopia 2020 (n = 422).

Among 28 women who reported symptomatic prolapse, 25 (5.9% of total) had anatomical prolapse when defined by vaginal checkups and confirmed by doctors. Thus, the prevalence of symptomatic prolapse and anatomical prolapses were 6.6% and 5.9%, respectively. Twenty-five women, 89.29% of those who reported symptoms of UP, had anatomical prolapse. The overall rate of UP was 5.9% (Fig 2).

### Factors associated with uterine prolapse

Multivariate logistic regression conducted identified, age at first marriage, history of abortion, birth attendant who assisted the delivery, and place of delivery were independent factors associated with uterine prolapse (p < 0.05).

Respondents who had a history of abortion were 2.94-times more likely to experience UP (AOR 2.94, 95%CI 1.08, 7.97). Women who were married at age 18 and above were 75% less likely to have uterine prolapse than those who were married before 18 years of age (AOR 0.25, 95%CI 0.07, 0.89). Home delivery was also a risk factor for UP, with women who had a history of home delivery 3.33-times more likely to have UP than other modes of delivery(AOR 3.33, 95%CI 1.21, 9.13). Moreover, women whose delivery was attended by a health professional were 79% less likely to have uterine prolapse than all other birth attendants (AOR 0.21; 95%CI 0.06, 0.71) (**Table 4**).

**Table 4:** Factors associated with the prevalence of uterine prolapse in the Loma Woreda, Dawuro Zone, Ethiopia 2020 (n = 422).

Variable	Category	Uterine prolapse		COR^^^ (95%CI)	P-Value	AOR (95%CI)	P-value
		Yes	No				
Residence	Urban	9	84	0.47 (0.21,1.12)	0.08	0.51 (0.21,1.28)	0.152
	Rural	16	313	1		1	
Educational status	Able to read and write	3	88	1		1	
	Unable to read and write	22	309	2.08 (0.61,7.14)	0.24	1.15 (0.28,4.66)	0.836
Age at first mirage	Less than 18 years	22	230	1		1	
	18 and above years	3	167	0.199 (0.05,0.64)	0.007	0.25 (0.07,0.89)	0.033*
Place of delivery	Home delivery	9	208	1.95 (0.84, 4.53)	0.117	3.33 (1.21,9.13)	0.020*
	Health institution	4	118	1		1	
Birth attendant	Health personnel	4	137	0.36 (0.12, 1.07)	0.067	0.21 (0.06,0.71)	0.011*
	Others	21	260	1		1	
History of abortion	No	18	361	1		1	
	Yes	7	36	3.9 (1.52, 9.96)	0.004	2.94 (1.08,7.97)	0.034*

*Significant at p-value<0.05

^ Crude odd ratio.

## Discussion

In this study, age at first marriage, a history of abortion, birth attendant who assisted the delivery, and place of delivery were independent factors associated with uterine prolapse.

Here we report the prevalence of symptomatic prolapse in Ethiopia, which was similar to global estimates (3–6%) with similar methodologies [3]. However, our prevalence of 5.9% was less than another study conducted in Nepal, which reported a 13% prevalence [13].

In Iran, the prevalence of UP among women of childbearing age has been reported to be 53.6%, significantly higher than our findings, although both reports early marriage and high parity as the strongest predictors of UP [14]. This significant difference in prevalence might be due to socio-cultural, ethnic, or racial differences. Similarly, the prevalence of UP among married women aged 15–60 in Lebanon was 49.6% [14], which again might be due to socio-cultural, ethnic, or methodological differences. A study from Gambia, West Africa, reported a prevalence rate of 46%, again with parity the strongest risk factor [4]. Besides, a study from Tanzania reported a 64.6% prevalence of anatomical prolapse, substantially higher than the 5.9% anatomical prolapse reported here [8]. However, both studies share home delivery as a risk factor.

Our findings are similar to those from Dabat, Northern Ethiopia, reporting a prevalence of symptomatic prolapse of 6.3% [13], although this study examined the prevalence in all women aged 18 and older rather than reproductive age women alone. Thus, our detected prevalence of UP seems high, given that the prevalence of UP increases with age [5, 13]. Moreover, our detected prevalence of UP is considerably higher than that reported in a study from North and East Ethiopia of only 1% [5]. The difference may be due to socio-cultural variations as well as methodological differences. In Keresa, Eastern Ethiopia, the prevalence of UP in ever-married women was 9.5% [15], a bit higher than reported in the current study.

The findings of this study and a similar study conducted in Wolaita Sodo of south Ethiopia showed age at first marriage and place of delivery were significantly associated with UP [16].

Intensive intermediate Obstetric Critical Care is needed to address maternal complications and their sustainability low resource setting. Studies conducted in Sierra Leone showed an additional cost per QALY of only €10.0; the implementation and one-year running of the case studied obstetric a highly cost-effective innovation [17]. Cost-effective, proven Obstetric Critical Care is needed to address maternal complications.

The limitation of the study was that only symptomatic uterine prolapses were included in this study.

## Conclusion and recommendations

UP is common in reproductive age women in Loma Woreda, Dawuro Zone, Ethiopia. Age at first marriage, place of delivery, birth attendant-assisted delivery, and history of abortion were found to be independent predictors of UP.

We recommend that the health system link primary health care to hospital-set for uterine prolapse treatment programs. Health institution delivery should be encouraged by the local government. Early marriage and unwanted pregnancy need to be prevented through appropriate strategies.

## Supporting information

S1 Data (SAV)

## Abbreviations

ANC: Antenatal Care
BMI: Body Mass Index
POP: Pelvic Organ Prolapse
UK: United Kingdom
UP: Uterine Prolapse
US: United States
USA: United States of America
UVP: Uterine Vaginal Prolapse
VAGH: Vaginal Hysterectomy

## References

[1] Shah P. Uterine prolapse and maternal morbidity in Nepal:. A human rights imperative Drexel L Rev. 2009;2:491.

[2] Parvathavarthini K, Vanusha A. Clinical epidemiological study of uterine prolapse. Int J ReprodContraceptObstetGynecol. 2019;8:7.

[3] Singh DR, Lama S. and Maharjan S. Knowledge on risk factors of uterine prolapse among reproductive age group women of Bajrabarahi Municipality of Lalitpur, Nepal. facilities. 2016; 6:1.

[4] Scherf C, Morison L, Fiander A., Ekpo G. and Walraven G. Epidemiology of pelvic organ prolapse in rural Gambia, West Africa. BJOG: An International Journal of Obstetrics &Gynaecology. 2002.;109(4):6. doi: 10.1111/j.1471-0528.2002.01109.x 12013164

[5] Megabiaw B, Adefris M., Rortveit G., Degu G., Muleta M., Blystad A., et al. Pelvic floor disorders among women in Dabat district, northwest Ethiopia: a pilot study. International urogynecology journal. 2013;24(7):8. doi: 10.1007/s00192-012-1981-y 23179499

[6] Thapa B, Rana G. and Gurung S. Contributing factors of utero-vaginal prolapse among women attending in Bharatpur hospital. Journal of Chitwan Medical College. 2014;4(3):5.

[7] Shrestha B, Onta S., Choulagai B., Paudel R., Petzold M. and Krettek A. Uterine prolapse and its impact on quality of life in the Jhaukhel–Duwakot Health Demographic Surveillance Site, Bhaktapur, Nepal. Global health action. 2015;8(1):1. doi: 10.3402/gha.v8.28771 26265389 PMC4532727

[8] Masenga GG, Shayo B.C.and Rasch V. Prevalence and risk factors for pelvic organ prolapse in Kilimanjaro, Tanzania: A population based study in Tanzanian rural community. PloS one. 2018;13(4):e0195910. doi: 10.1371/journal.pone.0195910 29694427 PMC5919002

[9] Karen Ballard FA, Jeremy Wright, Habtamu Atnafu. The prevalence of obstetric fistula and symptomatic pelvic organ prolapse in rural Ethiopia International Urogynecology Journal. 2016;27. 10.1007/s00192-015-2933-0 26755052

[10] Asresie A, Admassu E. and Setegn T. Determinants of pelvic organ prolapse among gynecologic patients in Bahir Dar, North West Ethiopia: a case–control study. p. International journal of women’s health. 2016;8:1. doi: 10.2147/IJWH.S122459 28003773 PMC5161336

[11] Elsayed F, Ahmed M. and Ahmed M.A.S. Knowledge and Practices of women regarding Risk Factors of Uterine Prolapse. IOSR Journal of Nursing and Health Science (IOSRJNHS). 2016;5(6):8.

[12] Awotunde OT, Fehintola A.O., Ogunlaja O.A., Olujide L.O., Aaron O.I., Bakare B, et al. An audit of uterovaginal prolapse in Ogbomoso, south-west Nigeria. Research Journal of Health Sciences. 2016;4(1):6.

[13] Silwal M, Gurung G., Shrestha N., Gurung A. and Ojha S. Prevalence and Factors Affecting Women with Uterine Prolapse in Lekhnath, Kaski, Nepal. 9(2), pp.52–57. Journal of Gandaki Medical College-Nepal. 2016;9(2):6.

[14] Neupane S. Effectiveness of self instructional module on level of knowledge regarding prevention and management of uterine prolapse among the perimenopausal women in selected urban area of Mangalore (Doctoral dissertation). 2013.

[15] Dheresa M, Worku A., Oljira L., Mengiste B., Assefa N. and Berhane Y. One in five women suffer from pelvic floor disorders in Kersa district Eastern Ethiopia: a community-based study. 18(1), p.95. BMC women’s health. 2018;18(1):1. doi: 10.1186/s12905-018-0585-1 29902997 PMC6003007

[16] ZinashLema Y, Mengistu Meskele. Determinants of Pelvic Organ Prolapse among Gynecological Cases in WolaitaSodo University Referral Teaching Hospital, Southern Ethiopia: A Case Control Study. Journal of Biology, Agriculture and Healthcare. 2015; Vol.5, No.21.

[17] Marotta C, Pisani L, Di Gennaro F, Cavallin F, Bah S, Pisani V, et al. Epidemiology, Outcomes, and Risk Factors for Mortality in Critically Ill Women Admitted to an Obstetric High-Dependency Unit in Sierra Leone. The American journal of tropical medicine and hygiene. 2020;103(5):2142–8. doi: 10.4269/ajtmh.20-0623 32840199 PMC7646769

