# Supplementary material for: Uterine prolapse and associated factors among reproductive-age women in south-west Ethiopia: A community-based cross-sectional study
Source: PLoS One. 2022 Jan 21;17(1):e0262077. doi: 10.1371/journal.pone.0262077 (PMC8782331; doi:10.1371/journal.pone.0262077)
Supplement: Attachment — Submitted filename: Response to reviewers_PLOS ONE.docx [file pone.0262077.s002.docx]

Date April 19, 2021.

PONE-D-20-40659
Uterine prolapse and associated factors among reproductive age women in Dawro Zone, southwest Ethiopia: a community based cross-sectional study
PLOS ONE

**Subject: Point by point response to reviewers' comments**

Dear PLOS ONE Editorial Manager,

Please kindly find submitted revised manuscript with ID number of PONE-D-20-40659, entitled “Uterine prolapse and associated factors among reproductive age women in Dawro Zone, southwest Ethiopia: a community based cross-sectional study” point to point response to reveiwers’ comment for kindly consideration.

With kind regards,

Abebe Sorsa (corresponding Author)

1. Please ensure that your manuscript meets PLOS ONE's style requirements, including those for file naming. The PLOS ONE style templates can be found at

Authors response: Dear Editor : Thank you so much, The manuscript meets PLOS ONE stylerequirements.

2. Please include additional information regarding the survey or questionnaire used in the study and ensure that you have provided sufficient details that others could replicate the analyses. For instance, if you developed a questionnaire as part of this study and it is not under a copyright more restrictive than CC-BY, please include a copy, in both the original language and English, as Supporting Information.

 Authors response: Dear Editor : Thank you so much, questionnaire developed included both original language and English, as Supporting Information.

3. Please amend your manuscript to adhere to our submission guidelines with respect to. Potentially, outmoded terms should be changed to more current, acceptable terminology. Specifically, we recommend that “Vaginal examination” should be changed to more appropriate term(s).

Authors response: Dear Editor : Thank you so much, “Vaginal examination” changed to vaginal checkup.

4. You indicated that you had ethical approval for your study. In your Methods section, please ensure you have also stated whether you obtained consent from parents or guardians of the minors included in the study or whether the research ethics committee or IRB specifically waived the need for their consent.

Authors response: Dear Editor : Thank you so much, No minors included in the study.

5. PLOS requires an ORCID iD for the corresponding author in Editorial Manager on papers submitted after December 6th, 2016. Please ensure that you have an ORCID iD and that it is validated in Editorial Manager. To do this, go to ‘Update my Information’ (in the upper left-hand corner of the main menu), and click on the Fetch/Validate link next to the ORCID field. This will take you to the ORCID site and allow you to create a new iD or authenticate a pre-existing iD in Editorial Manager. Please see the following video for instructions on linking an ORCID iD to your Editorial Manager account: <https://www.youtube.com/watch?v=_xcclfuvtxQ>

Authors response: Dear Editor : Thank you so much, I have an ORCID iD  0000-0002-1377-8298

6. Please amend your list of authors on the manuscript to ensure that each author is linked to an affiliation. Authors’ affiliations should reflect the institution where the work was done (if authors moved subsequently, you can also list the new affiliation stating “current affiliation:….” as necessary).

Authors response: Dear Editor : Thank you so much, list of authors amended on the manuscript.

7. Your ethics statement should only appear in the Methods section of your manuscript. If your ethics statement is written in any section besides the Methods, please move it to the Methods section and delete it from any other section. Please ensure that your ethics statement is included in your manuscript, as the ethics statement entered into the online submission form will not be published alongside your manuscript.

 Authors response: Dear Editor : Thank you so much, ethics statement included in the Methods section and deleted from other section.

8. We note you have included a table to which you do not refer in the text of your manuscript. Please ensure that you refer to Table 1 and 2 in your text; if accepted, production will need this reference to link the reader to the Table.

Authors response: Dear Editor : Thank you so much, we ensure that Table 1 and 2 refer in the text

9.We noticed you have some minor occurrence of overlapping text with the following previous publication(s), which needs to be addressed:

-<https://doi.org/10.1007/s00192-007-0375-z>

In your revision ensure you cite all your sources (including your own works), and quote or rephrase any duplicated text outside the methods section. Further consideration is dependent on these concerns being addressed.

Authors response: Dear Editor : Thank you so much, we revised and cited all sources.

**Reviewer #1**

I read the paper critically on an important topic and from a relevant setting. The paper is high quality: both statistical analysis and time of study is excellent some suggestion.

**Authors' response #2: Dear Reviewer, Thank you so much; reading critically and reviewing it and your suggestion.**

1. Introduction: UP is the most common gynaecological health problem contributing to maternal morbidity and mortality in women of reproductive age in developing countries. It is strongly related to infection pre and post-surgery. Infections are the underlying causes in 11% of maternal and one-fourth of newborn deaths, but the true burden of maternal infections and related complications remains unknown. Add and cite (Maternal caesarean section infection (MACSI) in Sierra Leone: a case-control study. Epidemiol Infect. 2020 Feb 27;148:e40. doi: 10.1017/S0950268820000370. PMID: 32102721; PMCID: PMC7058652.and Epidemiology, Outcomes, and Risk Factors for Mortality in Critically Ill Women Admitted to an Obstetric High-Dependency Unit in Sierra Leone. Am J Trop Med Hyg. 2020 Nov;103(5):2142-2148. doi: 10.4269/ajtmh.20-0623. PMID: 32840199; PMCID: PMC7646769.)

Authors' response: Dear Reviewer, Thank you so much; We included. "UP is the most common gynaecological health problem contributing to maternal morbidity and mortality in women of reproductive age in developing countries. It is strongly related to infection pre and post-surgery. Infections are the underlying causes in 11% of maternal and one-fourth of newborn deaths, but the actual burden of maternal infections and related complications remains unknown. (Di Gennaro et al., 2020, Marotta et al., 2020)."

Please see lines 69 to 73.

**Comment #2**. Methods and results: no comment are clear

**Authors' response #2: Dear Reviewer, Thank you so much;**

**Comment #3** Discussion: add and discuss the need for intensive care in Africa, especially to address maternal complications and sustainability, as highlighted in previous studies (see Cost-Utility of Intermediate Obstetric Critical Care in a Resource-Limited Setting: A Value-Based Analysis. Ann Glob Health. 2020 Jul 20;86(1):82. doi: 10.5334/aogh.2907. PMID: 32742940; PMCID: PMC7380057.)

**Authors' response to Comment # 3: Dear Reviewer, Thank you so much; We included "**Intensive intermediate Obstetric Critical Care is needed to address maternal complications and their sustainability low resource setting. Studies conducted in Sierra Leone showed an additional cost per QALY of only €10.0; the implementation and one-year running of the case studied obstetric a highly cost-effective innovation(Marotta et al., 2020)." Please see Linens 229 to 233.

Reviewer #2: The manuscript entitled "Uterine prolapse and associated factors among reproductive-age women in Dawro Zone, southwest Ethiopia: a community based cross-sectional study" is interesting, but I have some concerns:

**Authors' response** #2**: Dear Reviewer, Thank you so much; for your concerns**

**Comment #**1 - The manuscript title must be more objective, and other details must be mentioned in the methodology. We think the title "Uterine prolapse and associated factors among reproductive age women in southwest Ethiopia" would be better.

**Authors' response: Dear Reviewer, Thank you so much; we modified the manuscript title "Uterine prolapse and associated factors among reproductive-age women in southwest Ethiopia" Please see line 2 1ine 3.**

**Comment #** 2 - We suggest greater clarity of the Abstract, as it represents a mirror of the manuscript.

**Authors' response # 2: Dear Reviewer, Thank you so much; we clarity in the abstract of manuscript.**
**Comment #** 3 - In the Introduction, the authors write a long introduction with two pages and an unnecessary mention of the geographical situation and population of Ethiopia, it would be important to mention only the importance of the study in Ethiopia as it is an underdeveloped or developing country where in some regions there are lack of obstetric care.

**Authors' response# 3: Dear Reviewer, Thank you so much; we removed unnecessary mention of geographic situation."According to a projection from the 2007 national census, in 2019, Ethiopia has nearly 110 million inhabitants. The women in the reproductive age group constitute 23.4%. Thus, nearly 25.5 million Ethiopian women are between 15 and 49 years of age. These women, in one or other ways, are affected by the burden of uterine prolapse".**

**Comment #** 4 - This study lost international interest, as it was written with a view to more regional interest. We suggest that the authors review this situation.

**Authors' response#** 4: **Dear Reviewer, Thank you so much; we reviewed the situation.**
**Comment #** 5 - Also in Introduction or Discussion, the authors, despite studying uterine prolapse in reproductive age in an underdeveloped country, should stress that total uterine prolapse is more common in post-reproductive age, however carcinoma of the cervix and a prolapsed uterus are common diseases in underdeveloped countries , but their association is quite rare (da Silva BB. Carcinoma of the Cervix in Association with uterine prolapse. Gynecol Oncol
. 2002; 84 (2): 349-50. doi: 10.1006 / gyno.2001.6503).

**Authors' response# 5: Dear Reviewer, Thank you so much; we included in discussion part “Uterine prolapse is more common in post-reproductive age, however carcinoma of the cervix and a prolapsed uterus are common diseases in underdeveloped countries , but their association is quite rare (da Silva BB. Carcinoma of the Cervix in Association with uterine prolapse. Gynecol Oncol. 2002; 84 (2): 349-50. doi: 10.1006 / gyno.2001.6503).**

**Please see line 84 to 86.**

**Comment #** 6 - In Methods, why do the study on genital prolapse only in a community in Ethiopia with a smaller sample instead of studying with a larger sample size and greater international interest?

**Authors' response#6: Dear Reviewer, Thank you so much; we accept your comment; doing a study with a larger sample size and with greater international interest is more appropriate but due to financial constraints, we focused our study on genital prolapse.**

**We are planning to conduct an extensive study in future.**
**Comment #** 7 - In Results, we suggest greater objectivity, long and confusing results, occupying almost five pages.

**Authors' response#7: Dear reviewer; Thank you so much, we made results most concince and clean by removing all unnecessary statements with in three pages. Please see lines 160 to 203**
**Comment #** 8 - In discussion, we suggest that the authors discuss the findings of the current study, point by point, with findings in the international literature.

**Authors' response#8 : Dear reviewer; Thank you for your comment. We traid to to discuss point by point with finding from international literature.**
**Comment #** 9 - We suggest improving the English edition.

**Authors' response #9: Dear reviewer; Thank you so much. We made a language edition by international language editors—Nextgenediting for editorial assistance as part of their Global Initiative.**

**Reviewer #3:**

About the article "Uterine prolapse and associated factors among reproductive age women in Dawro Zone, southwest Ethiopia: a community based cross-sectional study":
First, I would like to congratulate the authors by this interesting paper. The authors' aim was to evaluate the prevalence and factors associated with uterine prolapse among women of reproductive age throw a cross-sectional study. The end point was to analyze risk factors associated with uterine prolapse.

**Authors' response: Thank you so much**
I have some points regard their work:
1- The article needs a native English speaker review;

**Authors' response#1: Dear reviewer, Thank you so much. We made language edition by international language editors. Nextgenediting for editorial assistance as part of their Global Initiative.**
2- The authors should describe the full words in first passage abbreviations (i.e. AOR, COR);

**Authors' response#2: Thank you so much. We fully desrcibed the full words in first passage abbreviation. Please see line 49.**
3- Introduction: The introduction is too long. The authors should shrink it. I suggest that the treatment section can be suppressed; repressed

**Authors' response#3: Thank you; we suppressed the treatment section. The treatment of UP depends on the severity of the prolapse, the woman's general health, age and desire to have children. First and second-degree prolapse is usually treated using ring pessaries inserted into the vagina to stretch the vaginal walls, often used in combination with pelvic floor exercises. The prolapse above the second degree is often treated by surgery (****Elsayed, 2016****).**

4- Introduction: last paragraph: I am not sure that they present the prevalence with this cross-sectional study. I understood that they analyzed a few sample of their population. How they make to this sample be representative? They should revise it;

**Authors' response#4: Thank you so much; we determined sample size based on scientific method size determination which gave us enough sample size. Participants were randomly selected in the study.**
5- Material and methods: "The sample size was calculated by using a single population formula with p=0.5 margin of error and (d) = 0.05". I couldn't understand how the authors calculated their sample size. What is their populational average used? And the standard deviation? Please, could you explain how it was estimated?;

**Authors' response#5: Thank you so much. We calculated sample size by using a confidence interval approach for a single population proportion with 50% since no published study regarding prevalence of UP in the country settled up. Therefore, we used to assume a proportion of 50% to get the maximum sample to be representative.**

6- Material and methods: How were the randomization performed?;

**Authors' response#6: Thank you so much. From the total 28 kebeles in the Loma district, 30% of kebeles, i.e., a total of eight kebeles, were selected by lottery method. The assumption was to divide the total estimated sample size to the households of each kebele according to the proportion they contribute to the total study subjects.**

**We allocated sample proportion for the selected eight kebeles based on sample size. Out of an estimated 422 participants, sample size was adjusted proportionally for the households of the selected eight kebeles. Then, households were selected by systematic random sampling method, i.e., selecting households at a fixed interval throughout a household list from a registration book of health post as provided by Health Extension Workers(HEWs) working at Health Post in each respective kebele**.
7- Material and methods: The authors describe that an interview done by midwives selected the patients? If they had symptoms, they were referred to the gynaecologist for a pelvic examination. The authors should describe as a weakness of the study that only symptomatic uterine prolapses were included in this study;

**Authors' response#7: Thank you so much. We described the li mitation of the study in the limitation of the study section. Please see lines 234 to 235.**
8- Material and methods: What questionnaire was used? What questions were used in each 3 main sections?;

**Authors' response#8: The interview question was composed of three main sections; the first two were phase 1 and the third was considered as phase two:**

**1) Socio-demographic variables and Obstetric and gynaecologic history (14 questions),**

**2) Questions regarding symptoms of uterine prolapsed (6questions),**

**3) The third section included two items; confirming by vaginal examination whether the women who reported symptomatic prolapse had anatomical prolapse or not, and the staging the prolapse was done then.**
9- Material and methods: Was the questionnaire validated by a previous study? Please indicate in the text the reference;

**Authors' response#9 : Thanks so much; we are not sure that the questionnaire was not validated in the previous study.**
10- Material and methods (abstract): I would like to understand why the authors included in the multivariate logistic regression the variables from the bivariate logistic regression with P < 0.25 instead of p<0.20. The authors should include this description in the Material and methods area of the main manuscript also;

**Authors' response #10: Thank you so much; We included variables with p< 0.25 for candidate variable for the final model to increase the chance of the variable became a significant predictor variable. We included the description in the material and methods section.**
11- Results: I would like to understand the results presented: "This study identified that the prevalence of symptomatic and anatomical uterine prolapse was 6.6% (28) and 5.9% (25) respectively". How can be more symptomatic patients that actually anatomical identified uterine prolapse? The authors should explain;

**Authors' response #11: Dear reviewer, thank you so much, the data collectors were trained midwifes and creafully assessed symptomatic patients and most of the symptomatic patients identified as anatomically identified utrine prolapse by doctors.**
12- Discussion: I suggest that the first paragraph, the authors describe their main objective findings;

**Authors' response #12: Dear reviewer, thank you so much, we included the statement “In this study; age at first marriage, a history of abortion, birth attendant who assisted the delivery, and place of delivery were independent factors associated with uterine prolapse.”**
13- Abstract Conclusion: The authors may enhance their conclusion. What is the message? What they suggest to their public health as a possible action?;

**Authors' response #13: Dear reviewer, thank you so much. We enhance the conclusion, including the message: We recommend that the health system link primary health care to hospital-set for uterine prolapse treatment programs. Health institution delivery should be encouraged by the local government. Early marriage and unwanted pregnancy need to be prevented through appropriate strategies. Please See line 49 to 52.**

14- Manuscript Conclusion: The authors should enhance the conclusion to better comprehension.
Cordially

**Authors' response #14: Dear reviewer, thank you so much. We enhance the conclusion to better comprehension, including the "We recommend that the health system is linking primary health care to hospital-set for uterine prolapse treatment programs. Health institution delivery should be encouraged by the local government. Early marriage and unwanted pregnancy need to be prevented through appropriate strategies." Please see lines 240- 243.**

Reviewer #4: 25/01/2021
Enclosed, please find a review of the manuscript entitled "Uterine prolapse and associated factors among reproductive-age women in Dawro Zone, southwest Ethiopia: a community based cross-sectional study" which I am recommending for publication in PLoS One after a minor revision. I have prepared a summary of the study and a list of issues that the authors may want to address
Title: "Uterine prolapse and associated factors among reproductive-age women in Dawro Zone, southwest Ethiopia: a community based cross-sectional study."
Comments for the author
General comments: I am happy of the chance to review this manuscript focusing on a subject that is still not adequately assessed, not only in developing countries but also in high income countries. I read the manuscript with interest and here is a list of minor concerns that the authors may want to address:
**Authors' response #: Dear Reviewer, thank so much**
1) I would suggest a linguistic revision in order to improve the readability of the manuscript
**Authors' response#1: Dear Reviewer, Thank you so much. We made a language edition by international language editors—Nextgenediting for editorial assistance as part of their Global Initiative**.
2) In the Methods section, the Authors report that variables with P-values less than 0.25 in bivariate logistic regression were further examined using multivariate logistic regression to investigate associations between the dependent variable and independent variables. How was this value of 0.25 chosen?

**Authors' response #2: Dear Reviewer, Thank you so much; We included variables with p< 0.25 for the candidate variable for the final model to increase the chance of the variable became a significant predictor variable. We included the description in the material and methods section.**
3) The Discussion section can be slightly extended to assess potential physiopathologic explanations for the associations found by the Authors.

**Authors' response #2: Dear Reviewer, Thank you so much; We extended the discussion part.**
