# Supplementary material for: Uterine prolapse and associated factors among reproductive-age women in south-west Ethiopia: A community-based cross-sectional study
Source: PLoS One. 2022 Jan 21;17(1):e0262077. doi: 10.1371/journal.pone.0262077 (PMC8782331; doi:10.1371/journal.pone.0262077)
Supplement: Attachment — Submitted filename: Response to Reviewers.docx [file pone.0262077.s004.docx]

Authors point by point response to Reviewer’s Responses to Questions

Reviewer’s Responses to Questions

**Comments to the Author**

1. If the authors have adequately addressed your comments raised in a previous round of review and you feel that this manuscript is now acceptable for publication, you may indicate that here to bypass the “Comments to the Author” section, enter your conflict of interest statement in the “Confidential to Editor” section, and submit your “Accept” recommendation.

Reviewer #1: All comments have been addressed

Authors’ response: Dear Reviewer, Thank you so much

Reviewer #3: (No Response)

2. Is the manuscript technically sound, and do the data support the conclusions?

The manuscript must describe a technically sound piece of scientific research with data that supports the conclusions. Experiments must have been conducted rigorously, with appropriate controls, replication, and sample sizes. The conclusions must be drawn appropriately based on the data presented.

Reviewer #1: Yes

Authors’ response: Dear Reviewer, Thank you so much

Reviewer #3: Partly

Authors’ response: Dear Reviewer, Thank you so much. We have revised the manuscript

3. Has the statistical analysis been performed appropriately and rigorously?

Reviewer #1: Yes

Authors’ response: Dear Reviewer, Thank you so much

Reviewer #3: No

Authors’ response: Dear Reviewer, Thank you so much; we have revised the manuscript

4. Have the authors made all data underlying the findings in their manuscript fully available?

The [PLOS Data policy](http://www.plosone.org/static/policies.action#sharing) requires authors to make all data underlying the findings described in their manuscript fully available without restriction, with rare exception (please refer to the Data Availability Statement in the manuscript PDF file). The data should be provided as part of the manuscript or its supporting information, or deposited to a public repository. For example, in addition to summary statistics, the data points behind means, medians and variance measures should be available. If there are restrictions on publicly sharing data—e.g. participant privacy or use of data from a third party—those must be specified.

Reviewer #1: Yes

Authors’ response: Dear Reviewer, Thank you so much

Reviewer #3: Yes

Authors’ response: Dear Reviewer, Thank you so much

5. Is the manuscript presented in an intelligible fashion and written in standard English?

PLOS ONE does not copyedit accepted manuscripts, so the language in submitted articles must be clear, correct, and unambiguous. Any typographical or grammatical errors should be corrected at revision, so please note any specific errors here.

Reviewer #1: Yes

Authors’ response: Dear Reviewer, Thank you so much,

Reviewer #3: No

Authors’ response: Dear Reviewer, Thank you so much; we utilized the nextgen professional editing for this manuscript and used Grammarly for checking it.

6. Review Comments to the author

Please use the space provided to explain your answers to the questions above. You may also include additional comments for the author, including concerns about dual publication, research ethics, or publication ethics. (Please upload your review as an attachment if it exceeds 20,000 characters)

Reviewer #1: In my opinion can be accepted, the paper is very good and also the time related to peer review is too much, and I think to make a decision in a short time, no one year.

Authors’ response: Dear Reviewer, Thank you so much for your time and reviewing this manuscript

Reviewer #3: About the revised article “Uterine prolapse and associated factors among reproductive-age women in southwest Ethiopia”: The authors’ aim was to evaluate the prevalence and factors associated with uterine prolapse among women of reproductive age throw a cross-sectional study. The endpoint was to analyze risk factors associated with uterine prolapse.
First, I would like to congratulate the authors by their effort in re-reviewing the reviewers’ statements. Each point with the revision needed (in my humble opinion):

Authors’ response: Dear Reviewer, Thank you so much for your time and reviewing this manuscript
1- The article needs a native English speaker review; ---- done

Authors’ response: Dear Reviewer, Thank you so much for time and reviewing this manucript
2- The authors should describe the full words in first passage abbreviations (i.e. AOR, COR);
“Authors’ response#2: Thank you so much. We fully desrcibed the full words in first passage abbreviation. Please see line 49.”------ Done.

Authors’ response: Dear Reviewer, Thank you so much for time and reviewing this manucript
3- Introduction: The introduction is too long. The authors should shrink it. I suggest that the treatment section can be suppressed; - done partially. The introduction remains too long. It would be better that they cut unnecessary parts. Authors’ response: Dear Reviewer, Thank you so much: we revised the introduction part.------ Done partially. The introduction remains too long. It would be better that they cut unnecessary parts.

Authors’ response: Dear Reviewer, Thank you so much; we revised the introduction part.

4- Introduction: last paragraph: I am not sure that they present the prevalence with this cross-sectional study. I understood that they analyzed a few sample of their population. How they make to this sample be representative? They should revise it; “Authors’ response#4: Thank you so much; we determined sample size based on scientific method size determination which gave us enough sample size. Participants were randomly selected in the study.” Thank you, but the answer is still lacking to me, as you can see the next question. Also, the authors described that the screening was done by trained midwives, finding 28 subjects that were suspicion of having UP. After doctor examination, only 25 confirmed. Also, the authors didn’t comment, but not even all UP the patients presents with symptoms. So, they can only estimate the incidence of symptomatic UP. The prevalence of UP is still not known, because only a few subjects were examined. Authors’ response: Dear Reviewer, Thank you so much: even though we determined sample size using the scientific method of sample size calculation the subjected examined for UP was few that affected the prevalence of UP among the reproductive age group.
--- Thank you, but the answer is still lacking to me. The answer has not clarified my previous questions. Also, the authors described that the screening was done by trained midwifes, finding 28 subjects that were suspicion of having UP. After doctor examination, only 25 confirmed. Also, the authors didn’t comment, but not even all UP the patients presents with symptoms. So, they can only estimate the incidence of symptomatic UP. The prevalence of UP is still not known, because only a few subjects were examined. Unfortunately, they do not know how many asymptomatic reproductive women have UP, so it is possible that their population prevalence be higher than they calculated;

Authors’ response: Dear Reviewer, Thank you so much. We estimated the prevalence of UP among the general reproductive age population of the Loma district. Even though the sample size was small, we determined the sample size using scientific methods.

5- Material and methods: “The sample size was calculated by using a single population formula with p=0.5 margin of error and (d) = 0.05 ”. I couldn’t understand how the authors calculated their sample size. What is their populational average used? And the standard deviation? Please, could you explain how it was estimated?; “Authors’ response#5: Thank you so much. We calculated sample size using a confidence interval approach for a single population proportion with 50% since no published study regarding the prevalence of UP in the country settled up. Therefore, we used to assume a proportion of 50% to get the maximum sample to be representative.” Thank you for the explanation. I am afraid that their explanation wasn’t enough to understand their sample size calculation. By the best of my limit knowledge, to calculate a sample by confidence interval approach for a single population proportion, you must know your standard deviation, not presented in the method area. Also, if they assume a 50% margin of error, that is a chance of 50% that their null hypothesis is true. The authors tried to use the sample calculation of other publications (Silwal M, Gurung, G., Shrestha, N., Gurung, A. and Ojha, S.: Prevalence and Factors Affecting Women with Uterine Prolapse in Lekhnath, Kaski, Nepal. , 9(2), pp.52-57. Journal of Gandaki Medical College-Nepal 2016, 9(2):6.). I suggest that they revise it with and epidemiologist or a mathematician. Authors’ response: Dear Reviewer, Thank you so much: to determine the maximum sample size for cross-sectional study; we determined sample size using a single population proportion formula with the assumption of the prevalence of UP among reproductive age 50%; p=0.5 and Z=1.96, Margin of error of 5% (d)= 0.05. we assumed a 5% of margin of error, not a 50%. A single population proportion gives a maximum sample size.
--------- Thank you. First I think you will have to correct the information in the material and methods. You answered here “Margin of error of 5% (d)= 0.05”; however, in the article you wrote “The sample size was calculated by using a single population formula with p=0.5 margin of error and (d) = 0.05 ”. Maybe your desire was to write: “The sample size was calculated by using a single population formula with a proportion of 0.5 and a margin of error of (d) = 0.05 ”
You assume a prevalence of 50%. You found around 6% of UP in your sample. You have estimated a high prevalence of UP compared with your sample results (6%). By the methodology used, your article was underpower. Also, there was already an evidence in Ethiopia of 22.3% (Silwal M, Gurung, G., Shrestha, N., Gurung, A. and Ojha, S.: Prevalence and Factors Affecting Women with Uterine Prolapse in Lekhnath, Kaski, Nepal. , 9(2), pp.52-57. Journal of Gandaki Medical College-Nepal 2016, 9(2):6.) that you could used to estimate your sample size.
If we consider that your sample calculation was adequate (to 6% of prevalence), your result is not a prevalence of 6%, because you have not diagnosticated women with asymptomatic UP. In my interpretation, you can conclude that the incidence of symptomatic UP of your sample was 5.9%.

Authors’ response: Dear Reviewer, Thank you so much for your time and reviewing this manuscript, perfectly we desired to write, “The sample size was calculated by using a single population formula with a proportion of 0.5 and a margin of error of (d) = 0.05 ”. we revised it. We used a proportion of 0.5 to get the maximum sample size. Suppose we would use prevalence UP evidence in Ethiopia of 22.3%, giving us a smaller sample size comparing using a proportion of 0.5. We intended to include a maximum sample size for our study even though the underpower.

6- Material and methods: How were the randomization performed?; “Authors’ response#6: Thank you so much. From the total 28 kebeles in the Loma district, 30% of kebeles, i.e., a total of eight kebeles, were selected by the lottery method. The assumption was to divide the total estimated sample size to the households of each kebele according to the proportion they contribute to the total study subjects. We allocated sample proportions for the selected eight kebeles based on sample size. Out of an estimated 422 participants, the sample size was adjusted proportionally for the households of the selected eight kebeles. Then, households were selected by systematic random sampling method, i.e., selecting households at a fixed interval throughout a household list from a registration book of health post as provided by Health Extension Workers(HEWs) working at Health Post in each respective kebele.” The explanation of randomization must be described in the “Material and methods” area. Authors’ response: Dear Reviewer, Thank you so much: we revised how was the randomization performed in material and methods sction. Please see lines 125 -133.
---- Thank you. If I understood right, you performed a simple random sampling, that is correct? If so, I suggest that you can say that you raffle 8 areas of Loma district and after that selected the sample by a simple random sampling in these areas. It will be less confusing to readers. I had to study what is a kebele. Thank you for the knowledge.

Authors’ response: Dear Reviewer, Thank you so much for your time: we revised it accordingly. Kebele is the lowest administrative unit in Ethiopia.
7- Material and methods: The authors describe that the patients were selected by an interview done by midwives? If they had symptoms, they were referred to the gynecologist to pelvic examination. The authors should describe as a weakness of the study that only symptomatic uterine prolapses were included in this study; Authors’ response#7: Thank you so much. We described the limitation of the study in the limitation of the study section. Please see lines 234 to 235. Done. I suggest that the authors present as a new paragraph only. It does not need that spotlight as a new subdivision in the discussion. Authors’ response: Dear Reviewer, Thank you so much: we revised it. Please see lines 256-257.
---- Thank you. The authors could present a paragraph with strengths and limitations of their work. They made only a final phrase of 1 limitation. They can cite that the article sample is underpower also, because of their prior prevalence estimation was more than 8 times higher than what they found.
Authors’ response#7: Dear Reviewer, Thank you so much for your time, we revised manuscript accordingly. Please see lines 238 – 240.
8- Material and methods: What questionnaires were used? What questions were used in each 3 main sections?; “Authors’ response#8: The interview question was composed of three main sections; the first two were phase 1 and the third was considered as phase two:
1) Socio-demographic variables and Obstetric and gynaecologic history (14 questions), 2) Questions regarding symptoms of uterine prolapsed (6questions),
3) The third section included two items; confirming by vaginal examination whether the women who reported symptomatic prolapse had anatomical prolapse or not, and the staging the prolapse was done then.” Thank you for the intent to answer the question. Unfortunately, the authors copy the explanation that was already in their article. The questions were not revealed in the article, neither in their review. It is not clear to readers what criteria was used to select patients. What questions were used? It is important to describe all the method used in the paper. Maybe their questions can be useful to others in the screening of UP. Authors’ response: Dear Reviewer, Thank you so much: the structured questionnaire used was included in the supplementary document section submitted with the manuscript.
-------- Thank you, I could understand the questionary used. Is there any reference of its use as a screening method to UP? Is it the first time used? If it is the first time used, I suggest that they validate it as a tool to population screening. In this case, all the sample must have gynecologic examination to rightly diagnose UP. The use of a non-validated questionary reinforces the question 5 (they could not conclude they population prevalence).

Authors’ response#8: Dear Reviewer, Thank you so much for your time. We reviewed different works of literature on similar studies and adopted and constructed the tool to screen UP.

9- Material and methods: Were the questionnaire validated by a previous study? Please indicate in the text the reference; “Authors’ response#9 : Thanks so much; we are not sure that the questionnaire was not validated in the previous study.” The question 9 is related to question 8. They do not show questions used. Also, they were not previous used in other articles. So, how they are so sure that their questions are adequate to screening of their sample? Authors’ response: Dear Reviewer, Thank you so much: we reviewed different literature of similar studies. We checked the validity of the questionnaire using Cronbach alpha that was greater than 0.7 that indicates the questionnaire measures what it intended to measure.
-------- Thank you. The Cronbach alpha is used to as a measure of internal consistency of a questionary, it is not used as a validation tool. That is OK, you already answered that the text was not previously validated in question 8. Who were the specialists that participated in the Cronbach alpha calculation? Were the authors of this article?

Authors’ response#9: Dear Reviewer, Thank you so much for your time. We reviewed different works of literature on similar studies and adopted and constructed the tool to screen UP. Pretest done ensure the reliability of the study tools and expert rating of the study tool done to ensure content validity of the tool by measuring the content validity index (CVI). Gynaecology experts, researchers were rated for the validity of the tool.
10- Material and methods (abstract): I would like to understand why the authors included in the multivariate logistic regression the variables from the bivariate logistic regression with P < 0.25 instead of p<0.20. The authors should include this description in the Material and methods area of the main manuscript also;
“Authors’ response #10: Thank you so much; We included variables with p< 0.25 for candidate variable for the final model to increase the chance of the variable became a significant predictor variable. We included the description in the material and methods section.”
Thank you for the explanation. Unfortunately, their desire to make variables more prone to be significant biased their study. I suggest that it be analyze again, with the correct parameters often used in medical literature.
Authors’ response: Dear Reviewer, Thank you so much: Biostaticians recommend using P < 0.25 as candidate variables in bivariate analysis for candidate variables for multivariate analysis.
--------- Thank you for your response. I am still confused. You describe on the abstract’s methodology area that you used variables with P-values less than 0.25 in bivariate logistic regression were further examined using multivariate logistic regression to investigate associations between the dependent variable and independent variables. However, in the methology’s area of the complete article, you do not describe it. Also, your tables did not show what variables was calculated with p<0.25 to use in the multivariable analysis. The authors should clarify or rectify the information.

Authors’ response#10: Dear Reviewer, Thank you so much for your time. We revised accordingly.
11- Results: I would like to understand the results presented: “This study identified that the prevalence of symptomatic and anatomical uterine prolapse was 6.6% (28) and 5.9% (25) respectively”. How can be more symptomatic patients that actually anatomical identified uterine prolapse? The authors should explain;
“Authors’ response #11: Dear reviewer, thank you so much, the data collectors were trained midwifes and creafully assessed symptomatic patients and most of the symptomatic patients identified as anatomically identified utrine prolapse by doctors.”
Thank you, unfortunately the question remains: how they find more symptoms than they find the pathology? The symptomatology is not a diagnosis. So, in the initial screening with midwifes, there were 28 subjects that were suspicion of having UP. After doctor examination, only 25 confirmed. They should make the corrections pointed.
Authors’ response: Dear Reviewer, Thank you so much; in the initial screening with midwives, there were 28 subjects that were suspected of having UP. After the doctor examination, only 25 were confirmed.
------- Thank you, I understood exactly that. So you agree that only 25 were confirmed as having UP. In that way, you cannot say that there were 28 patients with symptomatic UP. You must rectify the information in the text. Also, you cannot affirm that the prevalence of UP in your sample was 5.9%, because you do not know that (there are asymptomatic UP that were not included in your article).

Authors’ response#11: Dear Reviewer, Thank you so much for your time. We revised accordingly.

12- Discussion: I suggest that in the first paragraph, the authors describe their main objective findings;
“Authors’ response #12: Dear reviewer, thank you so much, we included the statement “In this study; age at first marriage, a history of abortion, birth attendant who assisted the delivery, and place of delivery were independent factors associated with uterine prolapse.”
------- Perfect done.

Authors’ response#12: Dear Reviewer, Thank you so much for your time.
13- Abstract Conclusion: The authors may enhance their conclusion. What is the message? What they suggest to their public health as a possible action?;
“Authors’ response #13: Dear reviewer, thank you so much. We enhance the conclusion, including the message: We recommend that the health system link primary health care to hospital-set for uterine prolapse treatment programs. Health institution delivery should be encouraged by the local government. Early marriage and unwanted pregnancy need to be prevented through appropriate strategies. Please See line 49 to 52.”
------ Done.

Authors’ response#13: Dear Reviewer, Thank you so much for your time.
14- Manuscript Conclusion: The authors should enhance the conclusion to better comprehension.
“Authors’ response #14: Dear reviewer, thank you so much. We enhance the conclusion to better comprehension, including the “We recommend that the health system is linking primary health care to hospital-set for uterine prolapse treatment programs. Health institution delivery should be encouraged by the local government. Early marriage and unwanted pregnancy need to be prevented through appropriate strategies.” Please see lines 240- 243.”
------- Done.

Authors’ response#14: Dear Reviewer, Thank you so much for your time.
15- Introduction: They erased lines 118-119: “According to a projection from the 2007 national census, Ethiopia had nearly 110 million 119 inhabitants in 2019, with 23.4% of women of reproductive age.”. The subsequent phase must be adjusted: “Given this massive potentially…”
Authors’ response: Dear Reviewer, Thank you so much; We revised it. Please see line 97.
------- Thank you. You have cutted the passage “According to a projection from the 2007 national census, Ethiopia had nearly 110 million 119 inhabitants in 2019, with 23.4% of women of reproductive age.” However, the subsequent phrase has lost its meaning. The authors should revise it.
Authors’ response#15: Dear Reviewer, Thank you so much for your time. We revised accordingly.
16- Discussion, lines 250-251: Symptomatic prolapse in your sample wasn’t 6.6%. If the patients had gynecological examination by doctors and they diagnosticated only 25 of the total sample of 422, your result is 5.9%. There are 3 patients screened to UP that had not the diagnosis of UP made.
Authors’ response: Dear Reviewer, Thank you so much; we revised it
------- Thank you. I already commented in the previous questions. Unfortunately, you have not reviewed.
Authors’ response #16: Dear Reviewer, Thank you so much for your time. We revised accordingly.Please see lines 219-220.
17- Figure 2: The same as the previous: the presence of uterine prolapse was only in 25 patients (5.9%). The authors should revise it. A questionary cannot be the gold standard to UP diagnosis. The clinical evaluation is the gold standard.
Authors’ response: Dear Reviewer, Thank you so much; we revised it
------- Thank you. I already commented in the previous questions. Unfortunately, you have not reviewed.

Authors’ response#17: Dear Reviewer, Thank you so much for your time. We revised accordingly.Please see figure 2.
18- Table 1: marital status: Is missing data of 1 patient. They should revise.
Authors’ response: Dear Reviewer, Thank you so much; we revised it
------ Done partially. The percentage is missing 0.2%
Authors’ response#18: Dear Reviewer, Thank you so much for your time. We revised
19- Table 1: religion: Is missing data of 1.7% of their sample. They should revise.
Authors’ response: Dear Reviewer, Thank you so much; we revised it
------ Done
Authors’ response#19: Dear Reviewer, Thank you so much for your time.
20 – Abstract, “The objective of this study was to assess the prevalence of and factors associated with uterine prolapse in women of reproductive age in Ethiopia.”: The objective was not accomplished by this study. UP can be asymptomatic. So, without a medical evaluation, it is not possible to determine the prevalence of UP in their population. The inclusion criteria were done by non-medical interview (using a questionary). The authors should revise it.
21 – Abstract, results, “The prevalence of symptomatic and anatomical uterine prolapse was 6.6% (28) and 5.9% (25), respectively”: Sorry, but the authors already said that 3 patients of the 28 symptomatic did not confirm the hypothesis of UP after medical examination. They did not revise the manuscript as previously indicated in question 04.
22 – Introduction, “UP is the most common gynecological health problem contributing to maternal morbidity and mortality in women of reproductive age in developing countries”: The authors should show the reference of their statement.
23 – Introduction: It is still too long. They should shrink it.

Authors’ response #23: Dear Reviewer, Thank you so much for your time. We revised it.
24 - The authors should describe the full words in first passage abbreviations (i.e. COR);

Authors’ response#24: Dear Reviewer, Thank you so much for your time. We revised
25 – The authors indexed the previous and the new revision. It was a little bit confusing to the identification of the new one.

Authors’ response#25: Dear Reviewer, Thank you so much for your time.
